# Age Differences in the Use of Written Materials During Healthcare Visits

**DOI:** 10.1093/geroni/igaf122.1400

**Published:** 2025-12-31

**Authors:** Tess Wild, Mark Lachs, Corinna Löckenhoff

**Affiliations:** Cornell University, Ithaca, New York, United States; Weill Cornell Medicine, New York, New York, United States; Cornell University, Ithaca, New York, United States

## Abstract

Prior to healthcare choices, people often search for information online, compile self-collected health data, or write down questions and priorities. Such patient-generated lists and agendas have potential benefits for streamlining healthcare decisions but may also derail patient-physician communication by introducing extraneous detail. It remains unclear whether older adults are more likely than younger adults to prepare written materials, as they are generally less likely to engage in pre-decisional information-seeking, but more likely to use external memory aids, and more likely to manage complex health conditions that would benefit from written documentation. To address this question, an adult lifespan sample (n = 457; Mage = 51.50; SDage = 19.00, aged 18-99 years) completed a pre-registered online survey assessing demographics, personality, health status, health-related attitudes, the timing of their most recent healthcare visit, and whether they brought written material to the visit. Logistic regressions revealed that those who brought written materials were significantly older (p < .01) and more likely to be male (p < .05). They also scored higher on neuroticism and reported more recent healthcare visits, a higher importance of being informed about their health, but a lower understanding of their health condition (all ps<.05). None of the other covariates showed significant associations with using written materials. Further research is needed to examine potential implications of older adults’ use of written materials for healthcare outcomes and patient satisfaction.

